# Microbiology in the Field: Construction and Validation of a Portable Incubator for Real-Time Quantification of Coliforms and Other Bacteria

**DOI:** 10.3389/fpubh.2020.607997

**Published:** 2020-11-25

**Authors:** Jordan Wight, Marie-Pierre Varin, Gregory J. Robertson, Yannick Huot, Andrew S. Lang

**Affiliations:** ^1^Department of Biology, Memorial University of Newfoundland, St. John's, NL, Canada; ^2^Département de Géomatique Appliquée, Université de Sherbrooke, Sherbrooke, QC, Canada; ^3^Wildlife Research Division, Environment and Climate Change Canada, Mount Pearl, NL, Canada

**Keywords:** Canadian lakes, freshwater, methods development, environment, *E. coli*

## Abstract

Performing microbiological assays on environmental samples in field settings poses logistical challenges with respect to the availability of suitable equipment or the ability to get samples to the laboratory in a timely fashion. For example, the viability of some bacteria can decrease greatly between sampling and arrival to the laboratory for processing. We developed and constructed rugged, reliable, and cost-effective portable incubators that were used by 10 independent field teams to perform microbiological assays on surface water samples from lakes across Canada. Rigorous testing and validation of our incubators ensured that incubation conditions were consistent within and across all 10 field teams and 2 sampling years. Samples from all sites were processed in duplicate and bacterial counts were highly repeatable within and across sampling teams. Bacterial counts were also found to be statistically equivalent to counts obtained with standard laboratory techniques using a conventional incubator. Using this method, thermotolerant coliforms (TTCs) and *Escherichia coli* were quantified from 432 lakes, allowing comparison to both historical datasets that relied on TTCs and those following current guidelines that use *E. coli* counts. We found higher loads at the shoreline than the middle of lakes and different patterns between ecozones. *E. coli* was not frequently detected, but many lakes exceeded Canadian guideline values for activities such as swimming and some even exceeded the guideline value for secondary recreational activities such as boating. To the best of our knowledge, this is the largest bacteriological water quality assessment of freshwater lakes to date in terms of both spatial scale and the number of lakes sampled. Our incubator design can be easily adapted for a wide variety of researcher goals and represents a robust platform for field studies and other applications, including those in remote or low-resources settings.

## Introduction

Surveillance and monitoring of bacterial contamination in aquatic environments is critical for public health for water recreation, consumption, and sanitation (1). Monitoring programs are in place for drinking water, wastewater, and for recreational beaches, but studying bacterial contamination in more remote locations poses many challenges (1). Microbiological analyses typically require a well-equipped laboratory with specialized equipment and expertise. Furthermore, the timely arrival of samples to testing laboratories is critical so that the microbial community in the sample changes minimally during transit (1–3). The viability of microbial cells can change drastically and inconsistently over time after collection of samples and this can lead to underestimation of microbial loads and results that do not accurately reflect the original sampled location (1–3). Various regulatory agencies indicate that samples for microbiological analysis should be processed within 24 h of collection, or up to 48 h for samples from remote locations, with the minimum holding time possible being preferred (ideally <8 h) (1–6). It is widely accepted when testing for many common indicator organisms that samples processed more than 24 h after collection poorly represent bacterial loads at the time of collection and make for inconsistent and unreliable results that can lead to inaccurate conclusions (1, 7).

Many monitoring programs for recreational areas sample on a daily or weekly basis (1, 8), however, these sampling and monitoring programs come at a large expense and are typically limited in geographic scope. Regulatory guidelines outline both the frequency of sampling and the minimum number of samples that should be collected for a given recreational area, allowing for only a limited regional inference. Guidelines often specify values for both the geometric mean of all samples and a single-sample maximum to account for the heterogeneous nature of water bodies (1, 7, 9). Therefore, any evaluation of microbiological water quality in areas that lack repeat sampling and long-term monitoring is considered to be only a snapshot of bacterial loads from that sampling location at that particular time (1).

To minimize temporal degradation, environmental samples would ideally be processed as soon as they are collected. Several studies have investigated the use of microbiological testing during field assessments and in low-resource settings (10–14), employing products such as Petrifilms™ (3M™). However, these studies often lacked a strong microbiological foundation and comparisons to standard techniques. Two separate field studies, one in Cambodia and another in Tanzania, compared the incubation of Petrifilms™ in a conventional 37°C incubator against ambient temperature incubation. Both studies found high rates of variability within and between the methods leading to low confidence in the results (12, 15). There is a clear need for the development of robust methods to incubate bacteria in the field leading to timely, reliable and reproducible quantification of bacteria in water samples (11, 14, 16–20).

A humanitarian outreach program by Engineers Without Borders (Austin, Texas) developed a low-cost, battery-operated incubator, the Armadillo, for use with Petrifilms™, with education about water quality in remote environments via the visually striking results being its primary use (14, 21). Similarly, a low-cost field incubator was used in an assessment of bacterial contamination of groundwater in Malawi (19). Although these designs improved greatly upon previous studies where samples were incubated at ambient temperature or by using body heat and securing them to their body, the lack of several important microbiological principals, measures of reliability and reproducibility of results, and comparisons with standard methods currently preclude these from being used for widespread research and monitoring activities. Although multiple previous studies have used portable incubators for sampling in remote locations, samples could only be incubated when the incubator was stationary, posing further limitations on their use (14, 17–20). These previous efforts have shown proof of concept for field-based microbiological testing using Petrifilms™ and highlight the demand for affordable and versatile portable incubators for field projects globally (14, 17, 19, 21, 22).

As part of a national lake assessment (23), we sought to obtain a snapshot of the state of bacterial contamination in lakes across Canada. In this endeavor, samples were collected across the country by field teams that were mostly working in remote locations and far from microbiological testing laboratories. We developed a method rooted in sound microbiological practices for culturing bacteria while in the field using rugged, reliable, custom-built portable incubators. Here we outline the construction, testing, and validation of these incubators, which can withstand the challenges of fieldwork. We highlight how this adaptable and highly reliable method, comparable to standard laboratory techniques, has been used for determining thermotolerant coliform (TTC) and *Escherichia coli* loads in Canadian lakes. This method was used by the NSERC Canadian Lake Pulse Network (LakePulse) (23) during the summers of 2018 and 2019 to determine bacterial loads in surface waters for 432 lakes across Canada.

## Materials and Methods

### Incubator Construction

**Figure 1:**
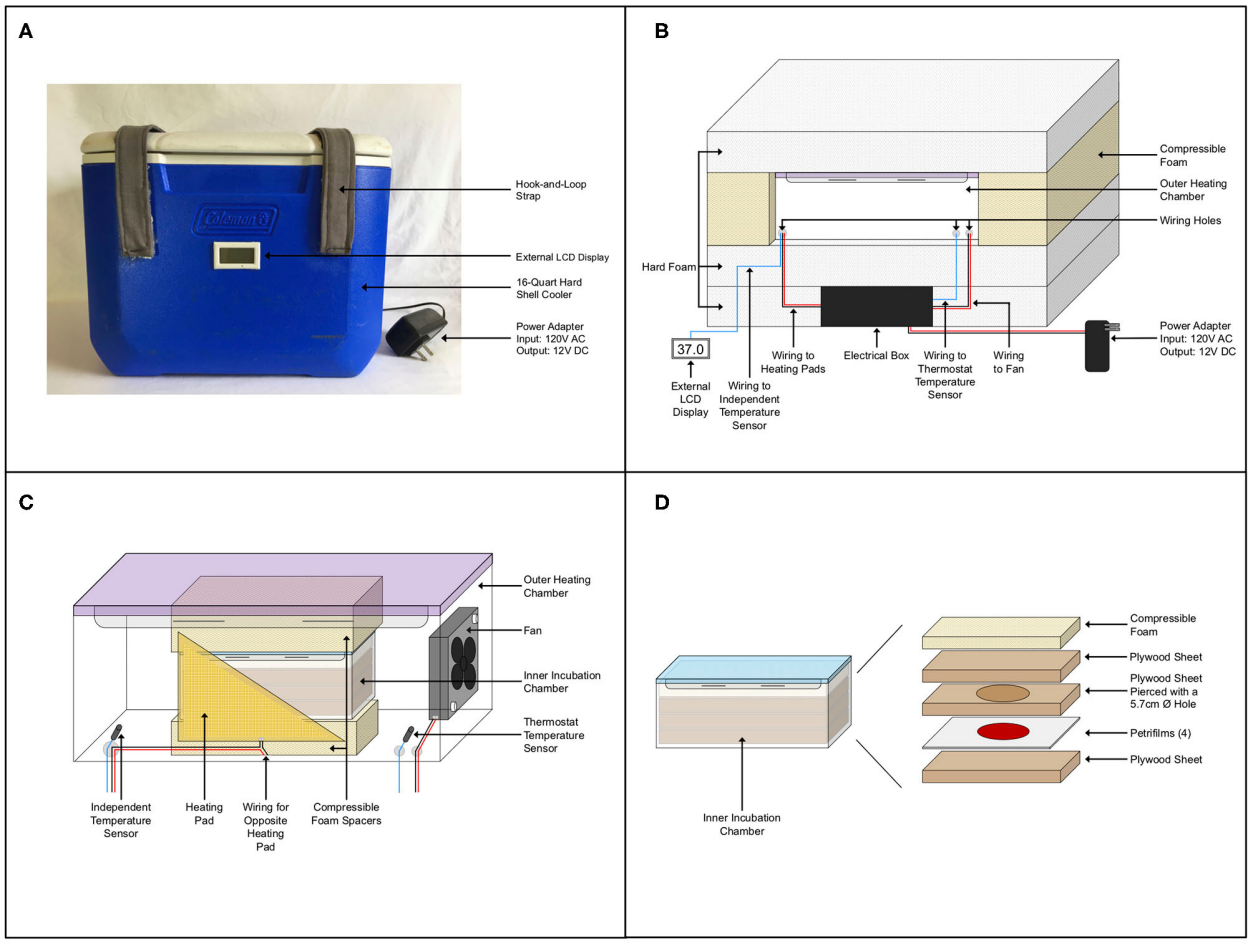
Incubator schematics. **(A)** Outside view of the incubator. **(B)** Internal layout of the incubator showing the electrical control system, wiring, foam insulation, and outer heating chamber. **(C)** Outer heating chamber detailed with wiring to fan and heating pads and foam spacers at the top and bottom of the inner chamber. Heating pads are rectangular, a cut-out is shown to see detail inside of the chamber. **(D)** Exploded view of the inner incubation chamber components.

The custom-built incubator featured a double-chamber design composed of two snap-lid leak-proof food storage containers inside of a 16-quart insulated hard-shell cooler (Figure 1). Hook-and-loop straps were attached to the front and top of the cooler to secure the lid during transport (Figure 1A). A 13/64″ hole was drilled in the back of the cooler for wiring. An acrylonitrile butadiene styrene (ABS) plastic junction box housed the electrical control system of the incubator and was secured to the inside of the cooler. This isolated the electrical system from the rest of the incubator and protected it during transport. Hard polystyrene foam R6.5 insulation was fit around the junction box and an additional layer above securely held the electrical components to the base of the cooler and gave a level surface on which to place the outer heating chamber. Compressible foam was fit around the heating chamber and an additional layer of polystyrene foam was placed on top of the chamber to prevent movement during transportation and to increase insulation (Figure 1B).

The outer heating chamber, a 3.3-L snap-lid leak-proof Tritan™ plastic food storage container, had three small holes drilled into it for wiring the two temperature sensors, two resistance heating pads, and a small axial fan (Figure 1C). The thermostat temperature sensor placed inside this chamber was connected to the thermostat control module. The thermostat regulated power sent to the heating pads that were taped to both long walls of the chamber, to allow maintenance of a constant temperature of 37°C. A small axial fan supplied with continuous power, offset by two spacers, was secured to one end to circulate heat throughout the chamber. A second independent temperature sensor secured inside this chamber was connected to an LCD display on the outside of the incubator so that the internal temperature could be monitored. The inner incubation chamber, a 0.52-L snap-lid leak-proof Tritan™ plastic food storage container, was raised to the middle of the outer chamber by compressible foam inserts at its top and bottom (Figure 1C). This allowed heat to circulate around the incubation chamber and ensured that it would not shift inside the heating chamber during transport.

Inoculated Petrifilms™ were stacked and placed on top of a piece of ½″ (1.27 cm) plywood that provided a level surface in the incubation chamber. A second piece of plywood with a 2 ¼″ (5.71 cm) hole, being slightly larger than the circular medium area of the Petrifilms™ (2″, 5.08 cm), was placed on top of the Petrifilms™. This was followed by another layer of plywood and a layer of compressible foam, securing the Petrifilms™ in place in the chamber (Figure 1D). The sandwiching of the Petrifilms™ in the incubation chamber ensured that the top film was not disturbed and that the medium area was not compressed. Petrifilms™ were secured in this way as the incubator was frequently moved, including during daily transport in the back of a truck while samples were incubating.

Several prototypes of the incubator were made throughout the design process, with the final version outlined here. In total, five incubators were constructed to these specifications and were used by the ten field teams, five teams per year, for the sampling campaigns of 2018 and 2019. While sampling in 2018, one team that occasionally experienced ambient temperatures below 15°C noticed that the incubator struggled to maintain 37°C. Prior to the field campaign of 2019, which extended up to 68° N in the Yukon and Northwest Territories, a layer of reflective bubble foil insulation was added to all inner sides of the incubators for added insulation. A full list of parts, materials, and tools used for construction of the incubators can be found in the Supplementary Material.

### Incubator Electrical System

Field teams had constant access to 120-volt AC power, supplied through a power converter from either a vehicle's electrical system, a portable generator, or a deep cycle battery bank, or through direct connection to standard electrical services. The power supply/adapter of the incubator converted this input power to 12-volt DC which was required for its electrical system. As incubators were supplied with continuous power, they were kept on for the entire duration of the 9 week field campaign. A schematic outlining the wiring of the incubator is shown in Supplementary Figure 1. The thermostat settings and a detailed description of the wiring are provided in the Supplementary Material.

### Incubator Testing

As the incubators were custom-built and used independently by different field teams, it was important to ensure temperature consistency within and between incubators. To evaluate this, a thermocouple was placed inside the outer heating chamber of each incubator, with all five thermocouples connected to a Campbell Scientific CR300 Datalogger. Following these tests, the set-point of the thermostat control module of the incubator was adjusted if necessary, to ensure that all incubators held the same internal temperature. The homogeneity of the temperature inside each incubator was evaluated by placing one thermocouple inside the inner incubation chamber, and the other four thermocouples in each corner of the outer heating chamber of one incubator. The time for the incubator to reach 37°C and the temperature stability over time were measured, with the change in temperature when disconnected from power also evaluated in 2019. In 2019, after adding a layer of reflective bubble foil insulation to the incubators, these tests were repeated at ambient temperatures of 4°C and at 24°C. A simulation of adding/removing Petrifilms™ was also performed in 2019, where the incubator was opened and the outer heating chamber and inner incubation chamber was disassembled for 1 min, to determine the time for the temperature to recover to 37°C. The power consumption of the incubator was determined by using a multimeter to measure the amperage when the heating pads were on and off. A logger recorded the time heating pads were on over a 12 h period, which allowed calculation of the mean amperage.

### Sampling Overview

The LakePulse survey involved five independent field teams each year. Each team was identified by a color (blue, green, purple, red, or yellow) and typically sampled one lake per day, in their respective regions, for 8 to 9 weeks during the summers of 2018 and 2019. Each lake was sampled once, for a total of 45 to 49 lakes per team per year.

Single sub-surface grab samples were aseptically collected in sterile 50-mL conical tubes (Fisher Scientific, Product # 05-539-13) at two sites per lake, one in the littoral zone and one above the deepest part of the lake, below referred to as the littoral and index sites, respectively. These were collected from 217 lakes in 2018 and 215 lakes in 2019. Samples were stored on ice or at 4°C for a maximum of 2 h before being processed.

### Sample Processing

*E. coli*/Coliform Plate Count Petrifilms™ (3M™) (Product # 6414) were used to determine TTC, *E. coli*, and non-coliform loads in water samples; the total number of colonies counted using this product is hereafter referred to as the total Petrifilm bacterial load. Samples were processed aseptically on a clean level table in a temporary outdoor laboratory, covered by a pop-up canopy tent. One person per field team was responsible for processing samples. Training by demonstration and with practice prior to the field campaign was provided to each responsible person. After shaking vigorously for 30 s, each sample was processed in duplicate as per the manufacturer's instructions. Petrifilms™ were incubated at 37°C for 24 ± 2 h. Following incubation, photos of the Petrifilms™ were taken and uploaded to an online server. An initial quality assurance step was made throughout both field campaigns by visually inspecting the photos to ensure labeling and processing protocols were followed by each team and that the quality of the photos was satisfactory.

### Interpretation and Quality Control of Results

After incubation, Petrifilms™ were removed from the incubator and backlit photographs were taken by the 8-megapixel camera of a 2017 iPad Pro (Apple, Toronto, Canada) while holding the Petrifilms™ up to the sky. Interpretation of the photographic results was later performed manually by a single individual as per the manufacturer's instructions, with the exception that all colonies were counted even if the number was above the recommended maximum countable range for the product. Photos were scored in a randomized order after assignment of a computer-generated randomized name to each JPEG image. After scoring, a random 10% of the images were re-scored to ensure consistency of results.

The data were subjected to several levels of quality control. Before analysis was performed, all samples that were not processed the same day of collection, were incubated beyond 24 ± 2 h, or contained algae or debris such as sediment that impaired scoring of results were removed. For any samples where colony counts differed by more than 10 colonies and/or >30% between duplicates, the photographs and/or the original incubated Petrifilms™, which were stored at 4°C after incubation, were consulted to verify the accuracy of any outliers and they were removed if necessary. Additionally, notes made by the individuals that processed the samples while in the field were consulted to flag and remove samples that may have been problematic. Prior to analysis, samples that had colony counts above the recommended maximum countable range of the product, >150 colony forming units per mL (CFU/mL) (24), were removed.

### Comparison to Standard Methods

A comparison to standard laboratory methods was performed to ensure that the data generated from this method were equivalent to those using a conventional laboratory incubator. Three sub-surface water grab samples were collected from a local lake and processed using the same protocol as outlined above. Samples were processed in triplicate and incubated in either a conventional laboratory incubator (Model No. 2005, VWR) or a custom-built incubator at 37°C for 24 ± 2 h. Scoring of results was performed and subjected to the same interpretation and quality control as outlined above.

### Data Analysis and Statistics

Data from the 2018 field season were used for method validation and preliminary analyses. R v3.5.0 (25) was used to perform statistical analysis using two different approaches and for data visualization using the packages ggplot2 v3.3.0 (26) and cowplot v1.0.0 (27). In all tests where a *p*-value was generated, *p* < 0.05 was considered as significantly different. On average, duplicate samples differed by a total Petrifilm bacterial count of 5.5 ± 8.2 colonies. Across the whole dataset, samples averaged a total Petrifilm bacterial count of 22.6 ± 31.1 CFU/mL.

For the first and traditional approach (4, 28), after bacterial colony counts of zero were removed, the data were log- (base 10) transformed to more closely follow a normal distribution (a log-normal distribution). To evaluate the reliability of replicates (precision), a precision criterion was calculated by preparing control charts (R charts) based on the range (*R*) of the log-transformed total Petrifilm bacterial loads between duplicates (4, 28). The mean range ($\bar{R}$) was then calculated. The precision criterion values were calculated for two different individuals on the same team, each individual team, and the compiled data of all teams. Briefly, the standard deviation of $\bar{R}$ was estimated by dividing $\bar{R}$ by the constant d_2_, which for duplicates (*n* = 2) is 1.128 (4). The estimated standard deviation was multiplied by three and added to $\bar{R}$ to determine the Upper Control Limit (*UCL*_*R*_), or the 99% confidence limit; this equation can be simplified as $\bar{R}$ multiplied by the constant D_4_ (3.27) (4). As we wanted to use the 95% confidence limit, or the Upper Warning Limit (*UWL*_*R*_), this value was calculated using the equation $UWL_{R}=\frac{2}{3}\left(D_{4}\bar{R}-\bar{R}\right)+\bar{R}$ (4, 28), which for duplicates reduces to the simplified equation $UWL_{R}=2.51\times \bar{R}$ [(29), as cited in (28)]. The calculated *UWL*_*R*_ value is hereafter referred to as the precision criterion. The lower the precision criterion value, the less variability there is between sample replicates, and as such, the greater the reliability. If the range of the log-transformed bacterial counts between duplicates is greater than the precision criterion, then the counts differ too much between replicates (i.e., above the 95% confidence limit) and the data should be investigated and potentially discarded (4, 28).

A precision criterion should be determined for each individual lab for each type of sample examined to determine what is acceptable reliability for that laboratory (4). As the incubators were custom-built and used by different field teams, the members of which largely changed between the 2018 and 2019 field campaigns, there were no previous data to determine the level of precision acceptable for this method for each field laboratory. Therefore, to evaluate reliability of replicates, if the precision criterion calculated between the two individuals on the same team or each individual team are similar, then the precision between the groups are also similar and the data can be considered to be reliable regardless of the individual or team.

For the comparison between the custom-built incubator and a conventional laboratory incubator, an F-ratio test was performed using the log-transformed total Petrifilm bacterial loads.

A drawback of the traditional approach using precision criteria is that bacterial colony counts of zero are eliminated due to the need for log transformation, therefore a second approach using more modern statistical methods was also used. As the data were based on bacterial colony counts and each sample was processed in duplicate, we used Generalized Linear Mixed Models (GLMMs) with negative binomial distributions and a log link function using the package lme4 v.1.1-23 (30) to fit our models. Depending on the analysis, fixed effects included in the models were individuals, teams, or incubators as factors. Sample duplicates were treated as random effects. Poisson distributions (where the mean is equal to the variance) were also considered, however some models indicated overdispersion (the variance exceeded and accelerated with the mean), indicating that negative binomial distributions should provide better fit. To calculate an index similar to traditional reliability from sample duplicates, we used the intra-class correlation coefficient (ICC) developed for GLMMs (specifically equation 3.10 for negative binomial models) (31). ICC values range from 0 to 1, with values close to 1 indicating that most of the variation in the data is coming from sources other than the replicate samples (i.e., there is little variation between the duplicate samples), while low values indicate that most of the variation is due to the differences between duplicate samples (32). Among-sample variance was extracted as the random effect of duplicate samples, while observation-level variance (analogous to the residual variance in linear models) was calculated using the log-normal approximation for negative binomial models (31) with the expectation of the mean calculated with equation 5.8 in Nakagawa et al. (31).

## Results

### Incubator Testing

**Figure 2:**
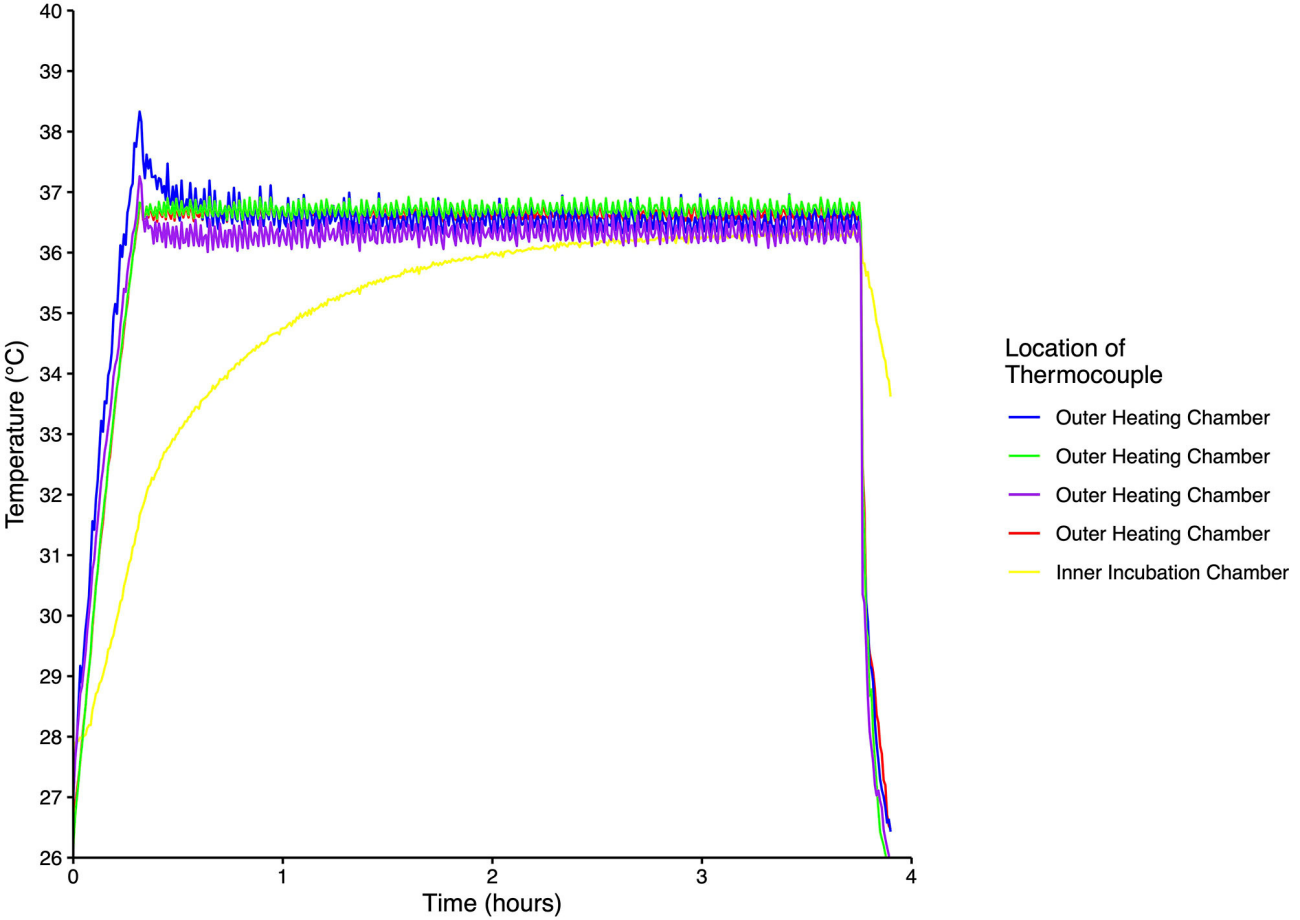
Temperature recordings from five thermocouples placed inside one incubator. Measurements were taken every 30 s for ~4 h at an ambient temperature of 26°C.

Temperature was homogeneous throughout the outer heating and inner incubation chambers for each of the five incubators and the temperature record for one of the incubators is shown in Figure 2. From a starting temperature of 26°C the temperature of the outer heating chamber stabilized after ~15 min, while the inner incubation chamber stabilized after ~125 min. Once the set temperature was reached, variability of both the outer and inner chambers was <1°C. When the incubator was turned off, the temperature of the outer chamber quickly decreased while the inner chamber temperature decreased at a much slower rate (Figure 2).

When tested at ambient temperatures of 26°C in 2018 and both 24 and 4°C in 2019, all incubators maintained a relatively constant temperature near 37°C during the testing period (Table 1, Supplementary Figure 2). The average temperature was 37.0°C in 2018, and 36.8°C when tested at 24 and 36.5°C when tested at 4°C in 2019 across all five incubators (Table 1). Although the temperatures were more variable at the lower ambient temperature, the range was always within 2°C (Table 1). When each incubator was tested at 4°C prior to the 2019 field campaign, the addition of the reflective bubble foil insulation enabled the incubators to maintain a constant temperature of 37°C ± 2°C. Under simulated conditions of adding/removing Petrifilms™ to the incubator, the internal temperature recovered in 14.2 min when at 24°C and 23.6 min when at 4°C (Table 1).

**Table 1:** Consistency of internal temperature profiles and recovery times after opening the incubator lid and disassembling the outer and inner chambers for 1 min.

**Ambient temperature (testing year)**	**Parameter^**a**^**	**Blue**	**Green**	**Purple**	**Red**	**Yellow**	**Average**
26°C (2018)	Average temperature (°C)	36.5	37.4	36.7	37.2	37.4	37.0
	Temperature range (°C)	36.2–36.9	37.0–37.9	36.4–37.1	37.0–37.5	37.1–37.9	36.2–37.9
24°C (2019)	Average temperature (°C)	36.6	37.1	36.8	36.6	37.2	36.8
	Temperature range (°C)	36.3–37.1	36.8–37.8	36.4–37.2	36.3–36.9	36.8–37.5	36.3–37.8
	Time to recover (minutes)	11	18	13	11	18	14.2
4°C (2019)	Average temperature (°C)	36.7	36.4	36.5	36.4	36.6	36.5
	Temperature range (°C)	36.1–37.6	35.7–37.3	35.7–37.4	35.5–37.4	35.8–37.5	35.5–37.6
	Time to recover (minutes)	19	25	23	25	26	23.6

a *Observations were made every 30 s and values were calculated from 23 and 64 h periods in 2018 and 2019, respectively; the time to recover was not tested in 2018*.

### Power Consumption

When the heating pads were on, the incubator drew approximately 1 A, and when the heating pads were off, the incubator drew ~0.1 A. For a period of 12 h at an ambient temperature of 24°C, the incubators consumed on average 2.9 W. When tested for the same period at an ambient temperature of 4°C, the incubators consumed on average 8.5 W.

### Statistical Analyses

To evaluate precision of replicates at the individual level, precision criterion-based analysis was used for a subset of the data, specifically Blue team. Due to a change-over in personnel part way through the 2018 field season, sample processing was performed by two different people over the course of the field season. After removing replicate pairs when either replicate had a count of zero, person A had a precision criterion of 0.357 (*n* = 31 paired samples), and person B had a precision criterion of 0.389 (*n* = 25). Precision criterion values at the team level were 0.371 for Blue team (*n* = 56), 0.393 for Green team (*n* = 47), 0.867 for Purple team (*n* = 35), 0.328 for Red team (*n* = 46), and 0.377 for Yellow team (*n* = 53), with an overall value of 0.442 for all teams (*n* = 237). All of these precision criterion values are relatively similar, although Purple team was least similar, indicating that there was relatively little variation between duplicate samples. Log-transformed total Petrifilm bacterial loads were not statistically different when samples were incubated in either a custom-built incubator or a conventional laboratory incubator (*F*_(1, 16)_ = 0.059, *p* = 0.811).

To evaluate precision of replicates at the individual level, the GLMM and ICC approach was used for Blue team's complete dataset (which retains zero values). Person A had an ICC of 0.99 (*n* = 42) and person B had an ICC of 0.99 (*n* = 34). ICCs at the team level were 0.99 for Blue team (*n* = 76), 0.99 for Green team (*n* = 58), 0.88 for Purple team (*n* = 63), 0.99 for Red team (*n* = 53), and 0.97 for Yellow team (*n* = 57), with an overall value of 0.99 for all teams (*n* = 307). All of these ICC values are close to 1, indicating that there was very little variation between duplicate samples. Using a negative binomial GLMM, mean total Petrifilm bacterial loads were not statistically different when samples were incubated in either a custom-built incubator or a conventional laboratory incubator (β = −0.102 ± 0.461, *p* = 0.825).

### Bacterial Loads and Lake Metrics

**Figure 3:**
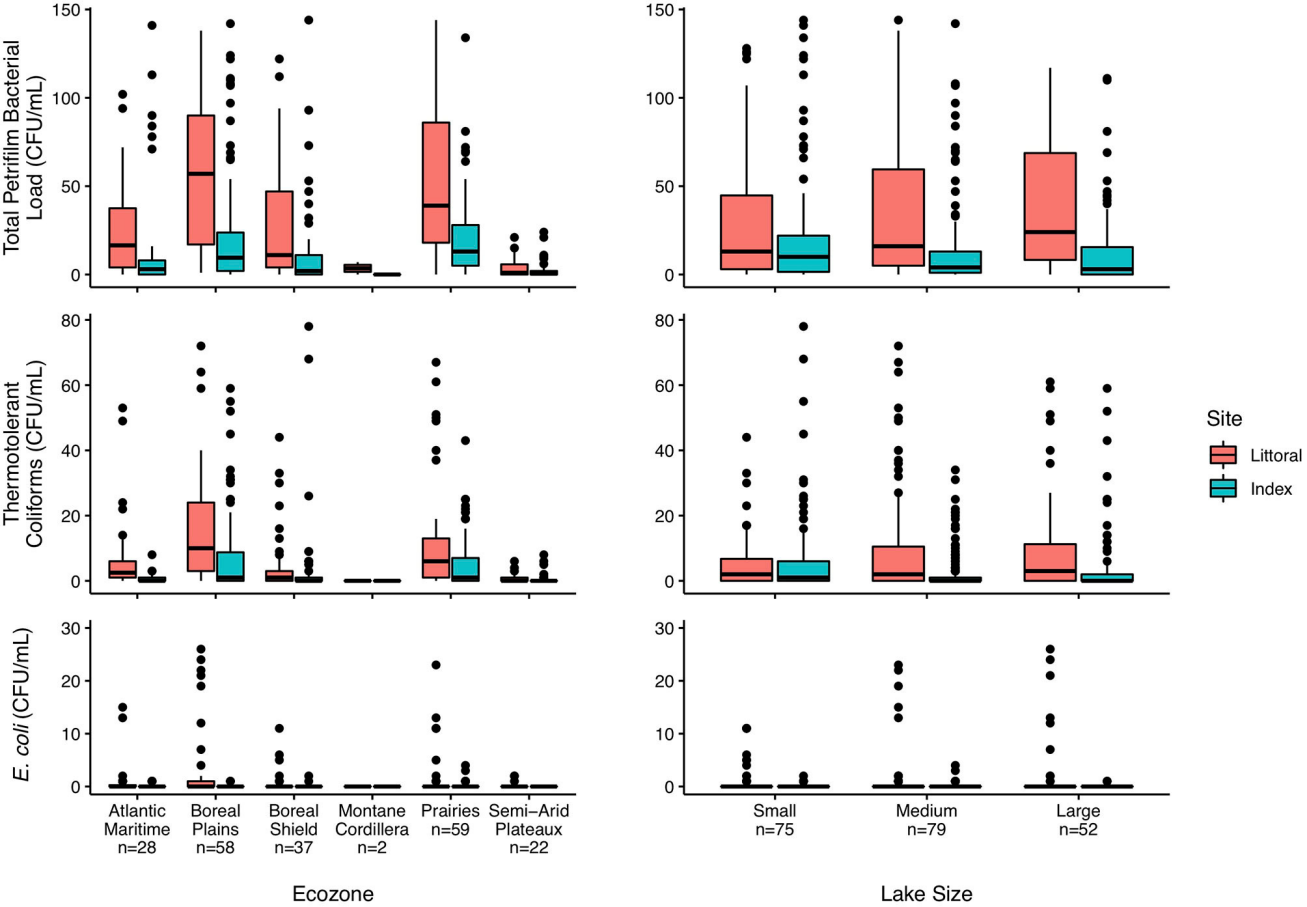
Boxplots of total Petrifilm, thermotolerant coliform, and *E. coli* counts segregated by lake ecozone and size. Centerlines show the median bacterial load, the upper and lower limits correspond to the first and third quartiles, with the whiskers showing the lowest and highest values within 1.5 times the interquartile range. Dots represent lakes with bacterial loads beyond the whiskers. Lakes were sampled in six of the 18 terrestrial ecozones in Canada, and lakes were classified by size, where small lakes are ≥0.1 to <0.5 km^2^, medium lakes are ≥0.5 to <5 km^2^, and large lakes are ≥5 to <100 km^2^ (23).

The relationship of bacterial loads and three lake metrics, ecozone, size, and sampling site, are shown in Figure 3. There was a wide range in bacterial loads present in these Canadian lakes. Across all ecozones and lake sizes, bacterial loads were higher at the littoral site than the index site. A large proportion of the bacteria cultured were non-coliforms. Similar trends at the ecozone and size levels were observed for TTC, with loads being higher in the Boreal Plains and Prairies ecozones and generally increasing with lake size (Figure 3). *E. coli* was not detected in the majority of the lakes; however, it was more common in lakes within the Atlantic Maritimes and Boreal Plains ecozones (Figure 3). There were no observable trends for *E. coli* loads and lake size.

## Discussion

Here we provide a framework for future researchers to build their own rugged, reliable, and cost-effective incubators that have been rigorously tested and whose design has been proven to withstand the challenges of field work while maintaining good microbiological standards.

### Incubator Validations

For a study of this scope and scale, rigorous testing and yearly validation of the custom-built incubators was of the highest importance. The temperature is homogeneous throughout the incubator due to the fan and double-chambered design. The fan helps to circulate heat in the outer heating chamber, minimizing hot spots nearest the heating pads and ensuring even heating on all sides of the inner incubation chamber. Temperature fluctuation is minimal, varying by <2°C from the setpoint. The temperature of the incubator recovers in a relatively short period of time after being opened for manipulations, as evidenced by monitoring during the simulated adding/removing of Petrifilms™, even at an ambient temperature of 4°C. Furthermore, the incubation chamber temperature decreases slowly when the incubator is briefly unplugged, further demonstrating its buffering capacity and ability to handle disruptions such as switching power sources (Figure 2).

The temperature performance of all five incubators was validated before each field season, ensuring consistent temperature conditions across all incubators and both sampling years. Testing in advance of the 2019 field campaign at 4°C, after adding the layer of reflective bubble foil insultation, showed that the incubators would be able to maintain temperature even below temperatures expected for Northern Canada in late August. Monitoring power consumption and periods of heating indicated that the incubators were adequately insulated for even a low-temperature environment. While in the field in 2018, several teams occasionally noticed that the internal temperature would rise above 37°C when the incubator was in direct sunlight on hot summer days. When incubators were moved out of direct sunlight, internal temperature would quickly return to 37°C. The level of insulation and the lack of cooling components were more than adequate for this Canadian field study and would similarly be for other studies in temperate climates. To the best of our knowledge, this is the first field-based study to perform both testing and validation of their incubator design.

### Data Validation

We performed statistical analyses using two different approaches for validation of our incubator and showed that data generated at the individual and team levels were comparable and reliable, despite one team (Purple) showing more variability. Purple team sampled lakes in the Semi-Arid Plateaux and Prairies ecozones where crop and livestock farming are common. Bacterial loads from their sampled lakes were frequently either below (<0.5 CFU/mL) or above (>150 CFU/mL) the detection thresholds. The approach where counts of zero were removed resulted in a smaller sample size for this team than for the other teams and this helps to explain why their precision criterion was much higher. This also inflated the overall precision criterion using data for all teams. Greater variability for this team was also shown using the second analytical approach, although it is much less pronounced. Model testing indicated that data from all other teams closely follow a Poisson distribution while Purple team's data were overdispersed, leading us to use negative binomial models for our analysis. These models include residual effects that are not included in Poisson models, and therefore ICCs for the other teams were more conservative than if a Poisson distribution was used, although they still show a very high level of reliability across all five teams.

Using the traditional approach, where zeros are removed and data are in log-transformed space, very low counts that differ by only a few colonies are often flagged as problematic (e.g., 4 and 10), although they are biologically acceptable. On the other hand, when counts are higher (e.g., 28 and 64), data are often shown to be acceptable using this approach, even though counts may differ by several fold and are not biologically acceptable. This statistical approach is often the standardized way in which the precision of duplicate analyses is determined by analytical laboratories as part of their quality assurance guidelines (4, 28). This type of analysis has significant limitations and poses several challenges for interpreting the data, largely due to log transformation. Therefore, in recent years it has been recognized and emphasized that other statistical approaches should be employed to evaluate precision (33, 34).

The more contemporary second approach takes into account the underlying distribution of the data and further highlights the limitations of using log transformation for count data. These contemporary analyses showed that each team had a high and comparable degree of reliability, something not as evident through the traditional log transformation approach. These findings not only support validation of our incubators but also show that the data are highly reliable and there are minimal artifacts in the dataset due to different processing individuals or teams/incubators. This statistical approach allows the data to be more useable for downstream analysis as counts of zero are retained and relationships of various factors with bacterial loads can be investigated directly (34).

### Relevant Microbiology

The direct plating method with a sample volume of 1 mL required when using Petrifilms™ is less representative than using a sample volume of 100 mL as frequently employed for methods such as membrane filtration and most probable number (MPN). Concentration of samples by membrane filtration was not feasible for this study as it would have resulted in the total bacterial loads being above the maximum countable range, and the results would also have been impaired by the presence of more algae and sediment on the filters. Although compact MPN methods exist that could be used for field applications, such as the IDEXX Quanti-Tray™, their size, cost, and the requirements for each team to have a tray sealer on site made this an unrealistic option for this study.

Although some of the sampled lakes are in urban and highly impacted environments, many of the lakes are minimally impacted by humans. Therefore, it is not surprising that TTC and *E. coli* loads were generally low. TTC loads show a much different trend than *E. coli* loads as TTC includes coliforms that are found both in the intestinal tracts of animals (fecal coliforms) as well as naturally in the environment. This also explains why they follow a similar trend as the total Petrifilm bacterial loads. An incubation temperature of 37°C was chosen for this study in order to determine TTC and *E. coli* loads. This allows for comparisons to the wealth of historical datasets that have used TTC as a measure of fecal contamination, as well as current datasets that follow guidelines based specifically on *E. coli* counts.

As the lower detection threshold for *E. coli*/Coliform Plate Count Petrifilms™ is 1 CFU/mL, or 100 CFU/100 mL, they are not sensitive enough for evaluation of bacteriological drinking water quality, which require a guideline value of 0 CFU/100 mL. However, they can provide the required bacteriological water quality information to determine if the waters are suitable for recreational activities. In Canada, guideline values for *E. coli* in fresh water for primary contact activities, such as swimming, are for a five-sample mean of ≤2 CFU/mL with a single sample maximum of ≤4 CFU/mL, while secondary contact activities, such as boating, have a guideline value of ≤10 CFU/mL (1). Most lakes sampled in 2018 were therefore of acceptable bacteriological water quality for activities such as swimming, whereas a few lakes exceeded the guideline value for secondary contact activities. More lakes in the Atlantic Maritimes and Boreal Plains ecozones had higher levels of *E. coli* than in the other ecozones, although most were still below the guideline value for secondary recreation. There appeared to be no trends with respect to lake size. A single grab sample at each site can show that fecal contamination has occurred, although, unlike using multiple samples along a given area, it provides only a single point snapshot of bacterial loads at that particular time.

### Applications and Potential Modifications

This field-based method allows bacterial loads to be accurately determined, including for very remote lakes, far away from traditional testing laboratories. By performing testing on-site within hours of collection, concerns about changes in the bacterial community between the time of sampling and the time of processing are minimized. This method can be used for a variety of future field studies when timely access to a microbiology laboratory is not possible and/or is cost-prohibitive. The incubator described here can be constructed in a matter of a few hours with minimal tools and expertise for a total materials cost of approximately $150 (CDN). Its components are readily available and can be easily purchased from hardware stores and online retailers. At a cost of approximately $2 (CDN) per Petrifilm™, bacterial loads of lake surface waters were determined for a total consumable cost of <$10 per lake. The low initial investment and consumable costs make this system highly cost-effective, and this approach can be used in the future by other research groups operating with modest budgets. The low-cost and flexibility for power supply options may also provide a solution for low- and middle-income countries with unreliable electricity to better perform microbiological testing and medical diagnostics in resource-limited settings (35, 36).

The methods used here can easily be adapted to suit the needs of future studies and are easily modified to work with many other microbiological testing products, including other products in the 3M™ Petrifilm™ suite. The incubator is inexpensive, easy to assemble, reliable, requires no specialized equipment, and is easily transported due to its ruggedness and low weight. It is easy to ship and travel with by air as there are no chemicals or batteries involved and no components are regulated as dangerous goods. For future research programs that do not have continuous access to electricity, the incubator design is easily modified to run on battery power. Modification to use an 8 Ah 12 V battery, which fits inside of the incubator, or a 100 Ah 12 V deep-cycle battery would power the incubator set at 37°C in an ambient temperature of 24°C for 33 h and 17 days, respectively, making it suitable for use in many different field scenarios.

## Conclusion

The design and validation of our incubator answers the call for the development of robust methods for performing bacteriological testing while in the field. Detecting *E. coli* in the surface waters of a lake raises concern as it highlights potentially significant fecal contamination, although this initial detection does not provide information on the source of the contamination (i.e., is it from humans, or wild or domesticated animals?). Further investigation into potential sources of the fecal bacteria are needed, taking into account surrounding land use variables and on-site observations during sampling, to identify the potential sources of contamination. Compilation of the datasets from both the 2018 and 2019 sampling campaigns will allow for a more comprehensive investigation into bacterial contamination in Canadian lakes, taking into account metrics such as the physicochemical properties of the lake water, surrounding land use characteristics, and potential impacts from wildlife.

## Data Availability Statement

The raw data supporting the conclusions of this article will be made available by the authors, without undue reservation.

## Author Contributions

YH and AL secured funding. JW, M-PV, YH, and AL conceived the study. JW and M-PV developed the methods and administrated the project. JW collected, processed, analyzed, and visualized the data. JW and GR carried out the statistical analysis. JW wrote the manuscript with contributions from M-PV. All authors read and revised the manuscript and gave final approval for publication.

## Conflict of Interest

The authors declare that the research was conducted in the absence of any commercial or financial relationships that could be construed as a potential conflict of interest.
