# Supplementary material for: Microbiology in the Field: Construction and Validation of a Portable Incubator for Real-Time Quantification of Coliforms and Other Bacteria
Source: Front Public Health. 2020 Nov 25;8:607997. doi: 10.3389/fpubh.2020.607997 (PMC7723852; doi:10.3389/fpubh.2020.607997)
Supplement: Supplementary file 1 [file Data_Sheet_1.PDF]

Supplementary Material

Supplementary Figure S1. Schematic of wiring of the custom-built incubators.

Supplementary Figure S2. Temperature recordings for all five incubators.

Supplementary Table S1. Specific products used for the construction of the incubator.

Supplementary Table S2. Additional material and tools required for construction of the incubator.

Supplementary Table S3. Settings for the Riorand thermostat controller panel.

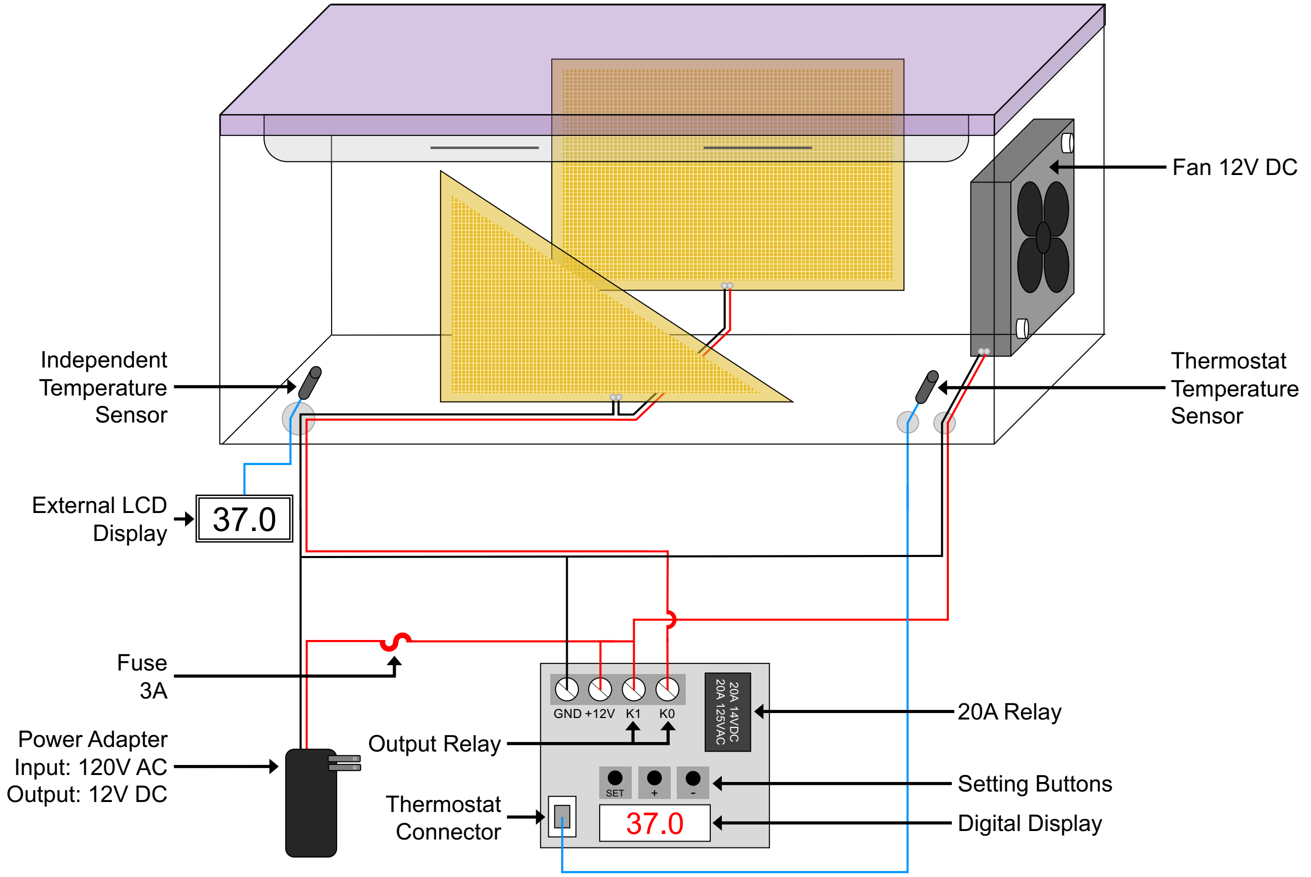

Supplementary Figure 1. Schematic of wiring of the custom-built incubators. A 3-amp fuse was connected to the positive wire from the power adapter. It was subsequently split into three positive wires that connected to 1) the axial fan, 2) the 12-volt DC power supply pole on the thermostat, and 3) the K1 output relay pole on the thermostat. A separate positive wire was connected to the K0 output relay; the relay between the K0 and K1 connectors open and close the electrical circuit based on the measured temperature in the outer chamber. The wire connected to the K0 output relay was connected to both heating pads in series that were attached to the outer chamber. The negative wire of the power supply was split into three wires and connected to 1) the negative wire of the axial fan, 2) the ground pole on the thermostat, and 3) the negative wire of the heating pads in series. Both heating pads are rectangular, pad in the forefront shown as a cut-out to see detail inside of the chamber.

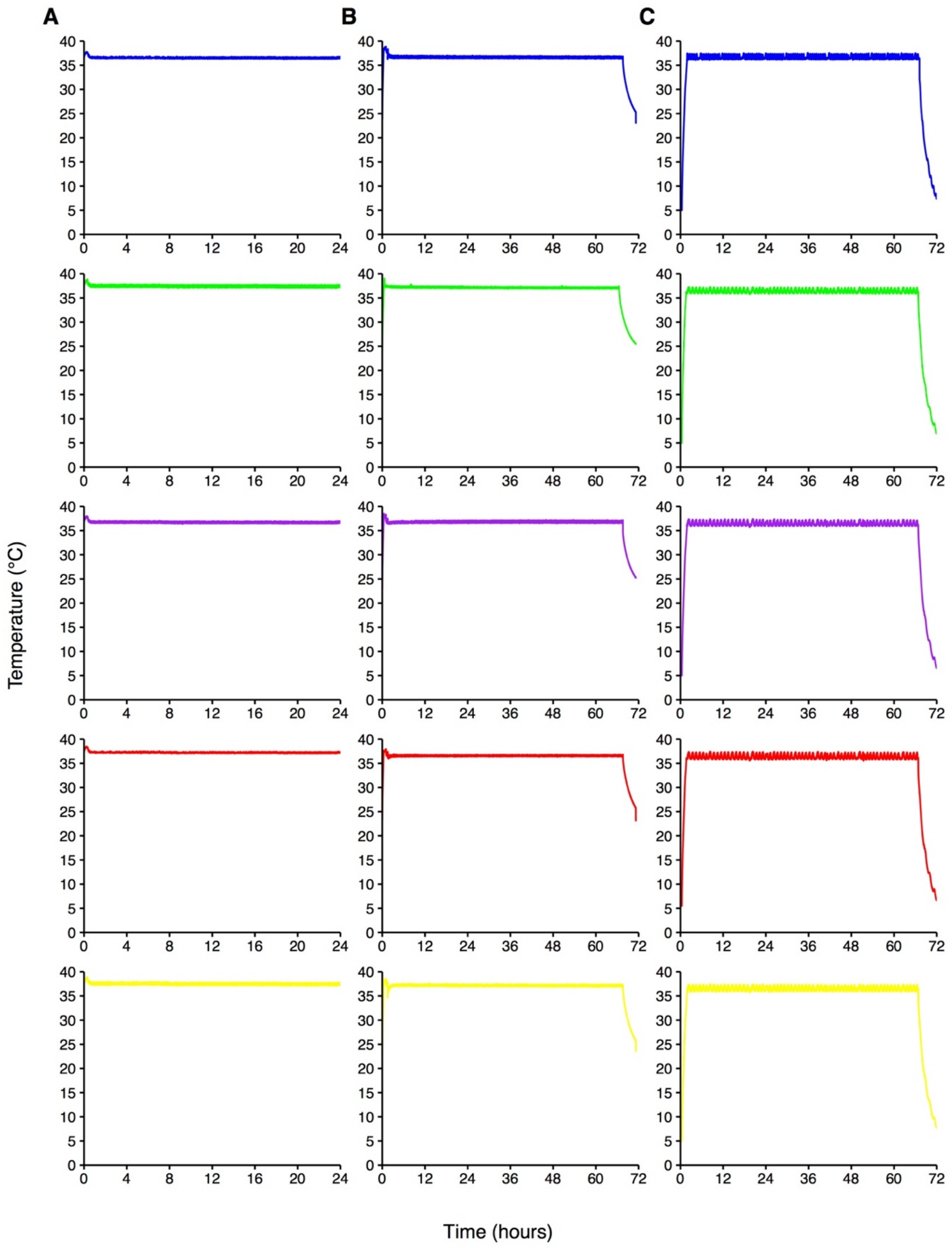

Supplementary Figure S2. Temperature recordings for all five incubators. Panel A shows the profile for each incubator, represented by the team colors, when tested in 2018 at an ambient temperature of 26 °C, while panels B and C show the profile for each incubator when tested in 2019 at an ambient temperature of 24 °C and 4 °C, respectively. Observations were taken every 30 seconds for 24 hours in 2018 and 72 hours in 2019.

Supplementary Table S1. Specific products used for the construction of the incubator.

Component	Brand	Model Number	Description	Cost (CAD)
Cooler	Coleman	3000001990	16-quart Excursion cooler	$49.00
Heating pads (2)	Sparkfun	COM-11288	5 x 10 cm heating pad	$5.53 (x2)
Thermostat controller panel	Riorand	B00KCCX0EE	12V digital temperature controller with waterproof probe	$13.99
Junction box	Abra Electronics	GPB324	Plastic box (5.75" L x 3.78" W x 2.3" H)	$6.99
Independent temperature sensor and display	Abra Electronics	TPM-10	LCD thermometer display with temperature Probe	$6.49
Fuse holder	Kolacen	KLFH-033	In-line 16-gauge fuse holder	$8.00
Batteries (2)	Energizer	LR44	Batteries for independent temperature sensor display	$2.00 (x2)
Power supply/adapter	Docooler	H8258	Power supply/adapter (AC 100-240 V to DC 12V 2A)	$13.00
Axial fan	Anvision	YDM4010B12	12V brushless cooling fan (40 x 40 x 10 mm)	$9.99
Food storage container (large)	Clearlock	3.3 L	3.3 L snap-lid leak-proof rectangular food storage container (26 cm L x 20 cm W x 9.5 cm H)	$10.99
Food storage container (small)	Lock & Lock	LBF311	520 mL snap-lid leak-proof rectangular food storage container (14 cm L x 10.5 cm W x 6 cm H)	$6.99
			Total	$140.50

Supplementary Table S2. Additional material and tools required for construction of the incubator.

Material and/or tools	Specification
Electrical wire	20-gauge
Electrical tape	-
Solder and soldering gun	-
Fuse	3 A
Heat shrink tubing	1/8” – 1/16”
Drill and drill bits	Various sized bits
Hole saw	2 ¼” diameter
Rotary tool	-
Wire strippers	-
Hard polystyrene foam	-
Compressible upholstery foam	-
Plywood	½” thick
Jig saw	-
Hook-and-loop straps	~6” long (2)
Glue gun	-
Reflective bubble foil insulation	-
Duct tape	-
Electrical outlet spacers	-
Screwdriver and screws	Various

Supplementary Table S3. Settings for the Riorand thermostat controller panel.

Control	Setting
Temperature	38.0°C
P0 (cooling/heating)	H
P1 (hysteresis)	0.1
P2 (highest setting limit)	50
P3 (lowest setting limit)	0
P4 (temperature correction)	0°C
P5 (delay start time)	0
P6 (key tone switch)	Off
